# Relationship between extravascular lung water and severity categories of acute respiratory distress syndrome by the Berlin definition

**DOI:** 10.1186/cc12811

**Published:** 2013-06-20

**Authors:** Shigeki Kushimoto, Tomoyuki Endo, Satoshi Yamanouchi, Teruo Sakamoto, Hiroyasu Ishikura, Yasuhide Kitazawa, Yasuhiko Taira, Kazuo Okuchi, Takashi Tagami, Akihiro Watanabe, Junko Yamaguchi, Kazuhide Yoshikawa, Manabu Sugita, Yoichi Kase, Takashi Kanemura, Hiroyuki Takahashi, Yuuichi Kuroki, Hiroo Izumino, Hiroshi Rinka, Ryutarou Seo, Makoto Takatori, Tadashi Kaneko, Toshiaki Nakamura, Takayuki Irahara, Nobuyuki Saito

**Affiliations:** 1 Division of Emergency Medicine, Tohoku University Graduate School of Medicine, 2-1, Seiryo-machi, Aoba-ku, Sendai, Miyagi 980-8575, Japan; 2 Department of Emergency and Critical Care Medicine, Tohoku University Hospital, 1-1 Seiryo-cho, Aoba-ku, Sendai, Miyagi 980-8574, Japan; 3 Department of Emergency and Critical Care Medicine, Kurume University School of Medicine, 67 Asahi-machi, Kurume-shi, Fukuoka 830-0011, Japan; 4 Department of Emergency and Critical Care Medicine, Faculty of Medicine, Fukuoka University, 8-19-1 Nanakuma, Jonan-ku, Fukuoka 814-0180, Japan; 5 Department of Emergency and Critical Care Medicine, Kansai Medical University, 10-15 Fumizonocho, Moriguchi, Osaka 573-1191, Japan; 6 Department of Emergency and Critical Care Medicine, St. Marianna University School of Medicine, 2-16-1 Sugao, Miyamae-ku, Kawasaki-shi, Kanagawa 216-8511, Japan; 7 Department of Emergency and Critical Care Medicine, Nara Medical University, 840 Shijo-cho, Kashihara, Nara 634-8521, Japan; 8 Department of Emergency and Critical Care Medicine, Nippon Medical School Hospital, 1-1-5 Sendagi, Bunkyo-ku, Tokyo 113-8603, Japan; 9 Department of Emergency and Critical Care Medicine, Nihon University School of Medicine Itabashi Hospital, 30-1, Oyaguchi Kami-cho, Itabashi-ku, Tokyo 173-8610, Japan; 10 Shock Trauma and Emergency Medical Center, Tokyo Medical and Dental University Hospital of Medicine, 1-5-45 Yushima, Bunkyo-ku, Tokyo 113-8510, Japan; 11 Department of Emergency and Critical Care Medicine, Juntendo University Nerima Hospital, 3-1-10 Takanodai, Nerima-ku Tokyo 177-8521, Japan; 12 Critical Care Medicine, Jikei University School of Medicine, 3-25-8 Nishi-shimbashi, Minato-ku, Tochigi 105 8461, Japan; 13 Emergency and Critical Care Medicine, National Hospital Organization Disaster Medical Center, 2-5-1, Higashigaoka, Meguro-ku, Tokyo 152-8902, Japan; 14 Department of Intensive Care Medicine, Saiseikai Yokohamashi Tobu Hospital, 3-6-1 Shimosueyoshi, Tsurumi-ku, Yokohama-shi, Kanagawa 230-8765, Japan; 15 Department of Emergency and Critical Care Medicine, Social Insurance Chukyo Hospital, 1-1-10. Sanjyo, Minami-ku, Nagoya, Aichi 457-8510, Japan; 16 Advanced Emergency and Critical Care Center, Kansai Medical University Takii Hospital, 10-15, Fumizono-cho, Moriguchi, Osaka 570-8507, Japan; 17 Emergency and Critical Care Medical Center, Osaka City General Hospital, 1-5-7 Asahimachi, Abeno-ku, Osaka 545-8585, Japan; 18 Intensive Care Unit, Kobe City Medical Center General Hospital, 4-6, Minatojimanakamachi, Chuo-ku, Kobe, Hyogo 650-0046, Japan; 19 Department of Anesthesia and Intensive Care, Hiroshima City Hospital, 7-33 Motomachi Naka-ku, Hiroshima-shi, Hiroshima 730-8518, Japan; 20 Advanced Medical Emergency and Critical Care Center, Yamaguchi University Hospital, 1-1-1, Minamikogushi, Ube-ku, Yamaguchi 755-8505, Japan; 21 Intensive Care Unit, Nagasaki University Hospital, 1-14 Bunkyo-machi, Nagasaki-ku Nagasaki 852-8521, Japan; 22 Department of Emergency and Critical Care Medicine, Nippon Medical School Tama Nagayama Hospital, 1-7-1 Nagayama, Tama-shi, Tokyo 206-8512, Japan; 23 Department of Emergency and Critical Care Medicine, Nippon Medical School Chiba Hokusou Hospital, 1715 Kamagari, Inzai-shi, Chiba 270-1694, Japan

## Abstract

**Introduction:**

The Berlin definition divides acute respiratory distress syndrome (ARDS) into three severity categories. The relationship between these categories and pulmonary microvascular permeability as well as extravascular lung water content, which is the hallmark of lung pathophysiology, remains to be elucidated. The aim of this study was to evaluate the relationship between extravascular lung water, pulmonary vascular permeability, and the severity categories as defined by the Berlin definition, and to confirm the associated predictive validity for severity.

**Methods:**

The extravascular lung water index (EVLWi) and pulmonary vascular permeability index (PVPI) were measured using a transpulmonary thermodilution method for three consecutive days in 195 patients with an EVLWi of ≥10 mL/kg and who fulfilled the Berlin definition of ARDS. Collectively, these patients were seen at 23 ICUs. Using the Berlin definition, patients were classified into three categories: mild, moderate, and severe.

**Results:**

Compared to patients with mild ARDS, patients with moderate and severe ARDS had higher acute physiology and chronic health evaluation II and sequential organ failure assessment scores on the day of enrollment. Patients with severe ARDS had higher EVLWi (mild, 16.1; moderate, 17.2; severe, 19.1; *P *<0.05) and PVPI (2.7; 3.0; 3.2; *P *<0.05). When categories were defined by the minimum PaO_2_/FIO_2 _ratio observed during the study period, the 28-day mortality rate increased with severity categories: moderate, odds ratio: 3.125 relative to mild; and severe, odds ratio: 4.167 relative to mild. On independent evaluation of 495 measurements from 195 patients over three days, negative and moderate correlations were observed between EVLWi and the PaO_2_/FIO_2 _ratio (r = -0.355, *P*<0.001) as well as between PVPI and the PaO_2_/FIO_2 _ratio (r = -0.345, *P *<0.001). ARDS severity was associated with an increase in EVLWi with the categories (mild, 14.7; moderate, 16.2; severe, 20.0; *P *<0.001) in all data sets. The value of PVPI followed the same pattern (2.6; 2.7; 3.5; *P *<0.001).

**Conclusions:**

Severity categories of ARDS described by the Berlin definition have good predictive validity and may be associated with increased extravascular lung water and pulmonary vascular permeability.

**Trial registration:**

UMIN-CTR ID UMIN000003627

## Introduction

Since the first description of acute respiratory distress syndrome (ARDS) by Ashbaugh *et al*. in 1967 [1], the definition had been continuously reworked until publication of the American-European Consensus Conference (AECC) definition in 1994 [2]. The AECC definition has been widely used for epidemiological studies, clinical trials, and critical care practice. It has facilitated advances in the acquisition of clinical and epidemiological data, leading to improvements in the care for patients with ARDS.

Although many clinical trials have been performed since publication of the AECC definition, several issues regarding the definition have emerged. These include a lack of explicit criteria for defining acute manifestations of the disease, sensitivity of the PaO_2_/FIO_2 _ratio (P/F ratio) to different ventilator settings, poor reliability of the chest radiograph criterion, and difficulties in distinguishing hydrostatic edema [3-6]. These criteria are also not sensitive predictors of disease severity or patient outcome [7-11].

Recently, theBerlin definition for ARDS has been published, focusing on feasibility, reliability, validity, and objective evaluation of its performance [12]. The definition includes mild, moderate, and severe ARDS based on the degree of hypoxemia. Progression from one category to another is associated with increased mortality.

ARDS is considered to be a type of acute, diffuse, inflammatory lung injury that leads to increased pulmonary vascular permeability, increased lung weight, and the loss of aerated lung tissue. However, no study has investigated the empirical relationship between a given ARDS stage and pulmonary microvascular permeability or extravascular lung water (EVLW) content [12].

Previous studies have reported various methods of quantifying pulmonary edema [13,14]. The double-indicator thermodilution technique allows researchers to measure the amount of EVLW. The *in vivo *and postmortem gravimetric EVLW values obtained using this method were closely correlated in both animal and human studies [15,16]. However, this method is excessively cumbersome and technically challenging for routine clinical application. Hence, the single-indicator technique is used in clinical settings; this method is as sensitive as the double-indicator technique [17,18]. Previously, we validated the accuracy of EVLW measurements obtained from postmortem lung samples using the single-indicator technique and defined statistically normal EVLW values, as determined through human autopsy [19]. The transpulmonary thermodilution technique provides an estimation of both EVLW and pulmonary blood volume. The ratio of these two parameters is denoted as the pulmonary vascular permeability index (PVPI). This ratio reflects the degree of pulmonary microvascular permeability [20], which is pathognomonic for ARDS.

The aims of this study were to evaluate the relationship between EVLW and pulmonary vascular permeability as assessed using the transpulmonary single thermodilution technique, on one hand, and severity categories of ARDS according to the Berlin definition in patients with acute respiratory failure, on the other. In addition, the study confirms the predictive validity of the Berlin stages with regard to disease severity and mortality.

## Materials and methods

This is a post hoc, subgroup analysis of a previously reported study conducted to clarify the clinical pathophysiological features of ARDS and to establish quantitative diagnostic criteria [21]. This study was approved by the ethics committee of each of the 23 institutions, and written informed consent was provided by each patient's next of kin. The investigation was registered with the University Hospital Medical Information Network (UMIN) Clinical Trials Registry, UMIN-CTR ID UMIN000003627.

### Patients

Between March 2009 and August 2011, 301 patients were enrolled. The inclusion criteria were: age ≥15 years; a need for mechanical ventilation (expected >48 h) for acute respiratory failure with a P/F ratio of ≤300 mm Hg; and bilateral infiltration as determined by chest radiography. The exclusion criteria were as follows: over five days from the onset of acute respiratory failure; chronic respiratory insufficiency; a history of pulmonary resection, pulmonary thromboembolism, severe peripheral arterial disease; a cardiac index of <1.5 L·min^-1^·m^-2^; lung contusion and burns; and other causes unsuitable for evaluation with the transpulmonary thermodilution technique.

**Figure 1:**
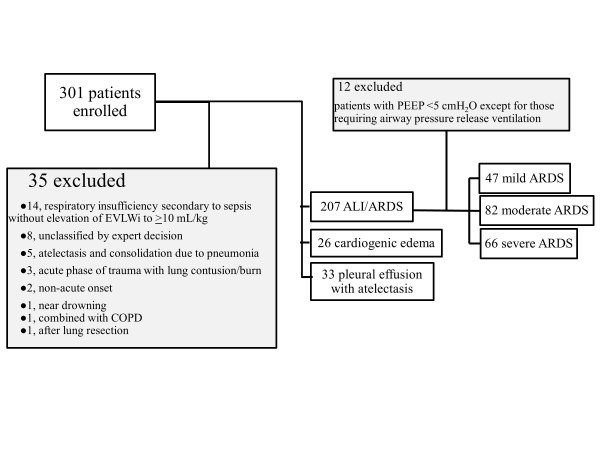
**Patient enrollment, exclusion, and classification**. ALI/ARDS, acute lung injury/acute respiratory distress syndrome; COPD, chronic obstructive pulmonary disease; EVLWi, extravascular lung water index; PEEP, positive end-expiratory pressure.

Among the 301 patients, 35 were excluded and 207 were considered to have acute lung injury (ALI)/ARDS (Figure 1).

The diagnosis of pulmonary edema was established on the basis of the following criteria: (1) the presence of bilateral infiltrates on chest radiographs; (2) a P/F ratio of ≤300 mm Hg; and (3) an increase in the EVLW indexed to the predicted body weight (EVLWi) of ≥10 mL/kg. Although there is no definitive quantitative criterion for the EVLWi indicative of pulmonary edema, we recently reported that the normal EVLWi value is approximately 7.4 ± 3.3 mL/kg in humans [19]. An increase in the EVLWi of ≥10 mL/kg was used to define pulmonary edema, as previously reported [22,23].

Among 207 patients determined to have ALI/ARDS, 12 patients with positive end-expiratory pressure (PEEP) <5 cmH_2_O who did not require airway pressure release ventilation were excluded. Each of the remaining 195 patients had experienced respiratory failure not fully explained by cardiac failure or volume overload and fulfilled the criteria of the Berlin definition. Based on the Berlin definition, patients/respiratory statuses were divided into three categories: mild, moderate, and severe ARDS (Figure 1).

### Measurement of *EVLWi *and pulmonary vascular permeability index

A thermistor-tipped catheter was connected to the PiCCO™ plus or PiCCO™ 2 monitor. This monitor uses a single-thermal indicator technique to calculate the cardiac output, global end-diastolic volume (GEDV), and EVLW. GEDV is calculated as the difference between the intrathoracic thermal volume (ITTV) and pulmonary thermal volume (PTV), which represents the combined end-diastolic volumes of four cardiac chambers. Intrathoracic blood volume (ITBV) was calculated as 1.25 × GEDV - 28.4 [17]. EVLW is the difference between the ITTV and ITBV [17,24]. PVPI was calculated as the ratio of EVLW to pulmonary blood volume [25-27]. The absolute EVLW value was indexed to predicted body weight [28-33]. The measurements of these parameters have been described in detail previously [21].

### Assessment of circulatory/respiratory status, other parameters, and clinical course

At the time of enrollment (day 0), the patient was evaluated with regard to clinical condition, cause of respiratory insufficiency, acute physiology and chronic health evaluation (APACHE) II score, sequential organ failure assessment (SOFA) score, and lung injury scale (LIS) score [34,35]. Echocardiography was performed to measure the left ventricular ejection fraction, left ventricular end-diastolic dimension, interventricular septum thickness, E/A ratio, left atrial dimension, inferior vena cava diameter and associated respiratory variation, the presence of hypo/akinesis, the presence of valvular abnormality, left ventricular systolic/diastolic function, and validity of the thermodilutional hemodynamic assessment. Chest computed tomography (CT) was also conducted on the day of enrollment. B-type natriuretic peptide (BNP) or *N*-terminal pro-BNP was measured on the day of enrollment and daily thereafter.

Circulatory/respiratory status was assessed from the day of enrollment to day 2. The patient's clinical course, including respirator setting, LIS score [36], SOFA score, antithrombin activity level, serum procalcitonin level, daily fluid intake/output and balance, and therapeutic interventions (surgery, antibiotics, steroids, diuretics, renal replacement therapy, and so on) were recorded daily. All patients were followed up for 28 days after enrollment.

### Determination of the pathophysiological diagnostic differential for respiratory insufficiency

At least three experts (specializing in intensive care, respirology, and cardiology) retrospectively determined the pathophysiological mechanism of respiratory insufficiency: (1) ALI/ARDS: increased pulmonary vascular permeability with or without increased pulmonary vascular hydrostatic pressure; (2) cardiogenic edema: increased pulmonary capillary hydrostatic pressure without increased vascular permeability; and (3) pleural effusion with atelectasis: no evidence of lung edema secondary to increased hydrostatic pressure or vascular permeability, as previously reported [25,37]. For this purpose, the experts carefully scrutinized the patient's medical history; clinical presentation and course; findings on chest CT, radiography, and echocardiography; concentrations of serum BNP or *N*-terminal pro-BNP and procalcitonin; and systemic inflammatory status. They also considered the time course of all the preceding findings, including daily fluid intake/output and the balance and requirement of systemic management and respiratory therapy. This pathophysiological diagnostic procedure was conducted by experts who were completely blinded to the PVPI findings.

### Statistical analysis

Data are presented as medians (interquartile range, IQR). Spearman's rank correlation was used for determining the correlation between variables, and Mann-Whitney's *U *test was used for assessing the differences between groups. For multiple-group comparisons, the analysis of variance on ranks was used with Tukey's test. The proportions were compared using Pearson's chi-square test. The odds ratios (95% confidence interval) are reported relative to a reference severity, defined as mild ARDS, for the risk of 28-day mortality. Receiver operating characteristic (ROC) curves were generated for lowest P/F ratio, highest PVPI and EVLWi by varying the discriminating threshold of each parameter and the area under the ROC curve for each parameter was calculated. A *P *value of <0.05 was considered significant. All statistical analyses were performed using SPSS 19.0 for Windows (SPSS, Chicago, IL, USA).

## Results

### Characteristics of patients with ARDS based on the Berlin definition on the day of enrollment

All 195 patients who presented with respiratory failure not fully explained by cardiac failure or volume overload, who also fulfilled the Berlin definition, were included. The patients were divided into the following three categories on the basis of their respiratory status on the day of enrollment: (1) mild ARDS, (2) moderate ARDS, and (3) severe ARDS. Table 1 shows the patient characteristics as measured on the day of enrollment.

**Table 1:** Characteristics of patients with ARDS based on the Berlin definition on the day of enrollment.

	mild ARDS (*n *= 47)	moderate ARDS (*n *= 82)	severe ARDS (*n *= 66)
Age, years	68 (59-78)	68 (59-82)	67 (53-78)
Gender (male), % (n)	70.2% (*n *= 33)	63.4% (*n *= 52)	66.7% (*n *= 44)
APACHE II score, points	19 (13-25)	25 (20-28)*	25 (18-30)*
SOFA score, points	9 (6-12)	11 (9-13)*	11 (10-14)*
SIRS criteria	2.0 (1.0-3.0)	2.0 (1.8-3.0)	3.0 (2.0-3.0)**
FIO_2_	0.5 (0.4-0.6)	0.6 (0.5-0.8)*	1.0 (0.8-1.0)*#
PEEP, cmH_2_O	8 (5-10)	10 (6-12)	10 (6-12)
PaO_2_/FIO_2 _ratio, mm Hg	247 (217-280)	148 (122-173)*	76 (60-90)*#
GEDI, mL/m^2^	780 (662-975)	814 (711-925)	730 (620-952)
EVLWi, mL/kg	16.1 (12.2-18.1)	17.2 (12.9-22.9)	19.1 (14.4-22.9)**
PVPI	2.7 (2.2-3.2)	3.0 (2.4-3.6)	3.2 (2.6-4.1)*##
Ventilator-free days within 28 days	18 (0-20)	9 (0-19)	4 (0-19)
28-day mortality, % (n)	23.4% (*n *= 11)	43.9% (*n *= 36)**	42.4% (*n *= 28)**

*P <0.01 vs. mild ARDS; **P <0.05 vs. mild ARDS; #*P *<0.01 vs. moderate ARDS; ##*P *<0.05 vs. moderate ARDS. ARDS, acute respiratory distress syndrome; APACHE, acute physiology and chronic health evaluation; SOFA, sequential organ failure assessment; SIRS, systemic inflammatory response syndrome; PEEP, positive end-expiratory pressure; GEDI, global end-diastolic blood volume index; EVLWi, extravascular lung water index; PVPI, pulmonary vascular permeability index.

There was no significant difference in age and gender associated with severity of ARDS. On the day of enrollment, both moderate and severe ARDS patients had higher APACHE II and SOFA scores than did patients with mild ARDS, and the positive number of systemic inflammatory response syndrome (SIRS) criteria was also higher in patients with severe ARDS as compared to those with mild or moderate ARDS. Although the levels of PEEP were similar among stages, the level of FIO_2 _required increased with ARDS severity. EVLWi and PVPI on the day of enrollment were significantly higher in severe ARDS patients. While 28-day mortality was higher in patients with moderate and severe ARDS than in patients with mild ARDS, there was no significant difference in the number of ventilator-free days within 28 days among patients with mild, moderate, or severe ARDS.

### ARDS severity as defined by minimal PaO_2_/FIO_2 _ratio and patient outcome

For this analysis, ARDS severity was determined based on the lowest P/F ratio measured during the three-day study period. At 28 days, the level of mortality was higher among patients with moderate or severe ARDS as opposed to those with mild ARDS. Severe ARDS was associated with fewer ventilator-free days than was mild ARDS (Table 2). Table 2 also shows the odds ratio for moderate and severe ARDS relative to mild ARDS: moderate ARDS, odds ratio: 2.824 (*P *= 0.048); severe ARDS, odds ratio: 4.167 (*P *= 0.003).

**Table 2:** Severity of ARDS defined by minimal PaO_2_/FIO_2 _ratio and outcome.

	mild ARDS (*n *= 33)		moderate ARDS (*n *= 83)	severe ARDS (*n *= 79)
Ventilator-free days within 28 days	18 (1-22)		9 (0-19)	0 (0-18)*

				

	28-day mortality	Unadjusted odds ratio	95% confidence interval	*P *value

mild ARDS (*n *= 33)	18.2% (*n *= 6)	1	(reference)	
moderate ARDS (*n *= 83)	38.6%** (*n *= 32)	2.824	1.050-7.590	0.048
severe ARDS (*n *= 79)	48.1%** (*n *= 38)	4.167	1.553-11.236	0.003

*P <0.01 vs. mild ARDS; **P <0.05 vs. mild ARDS. ARDS, acute respiratory distress syndrome.

The area under the curve to predict the outcome for the lowest P/F ratio (0.635; confidence interval, 0.556 to 0.715) was significantly larger than that for EVLWi (0.521; confidence interval, 0.437 to 0.605) and PVPI (0.533; confidence interval, 0.450 to 0.616) (*P *<0.05).

### Relationship among extravascular lung water, pulmonary vascular permeability, and ARDS severity

For this analysis, 495 sets of measurements from 195 patients obtained over the course of three days were independently evaluated and classified as mild, moderate, or severe ARDS according to the Berlin definition.

**Figure 2:**
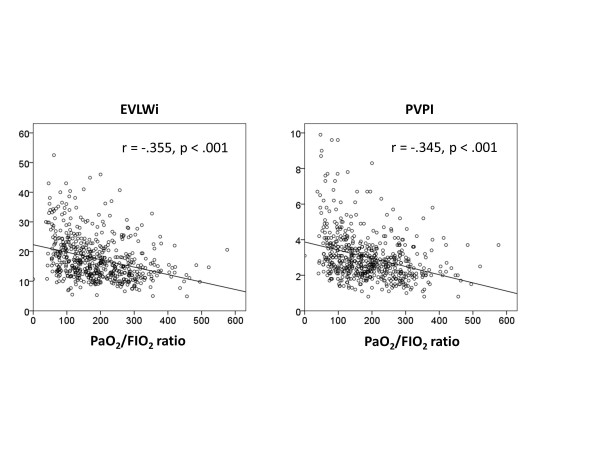
**Correlations among the extravascular lung water index, pulmonary vascular permeability index measured using transpulmonary thermodilution, and the PaO_2_/FIO_2 _ratio**. Negative and moderate correlations were noted between EVLWi and the PaO_2_/FIO_2 _ratio (r = -0.355, *P *<0.001) **(A)**, as well as between PVPI and the PaO_2_/FIO_2 _ratio (r = -0.345, *P *<0.001) **(B) **in all data sets. EVLWi, extravascular lung water index; PVPI, pulmonary vascular permeability index.

**Figure 3:**
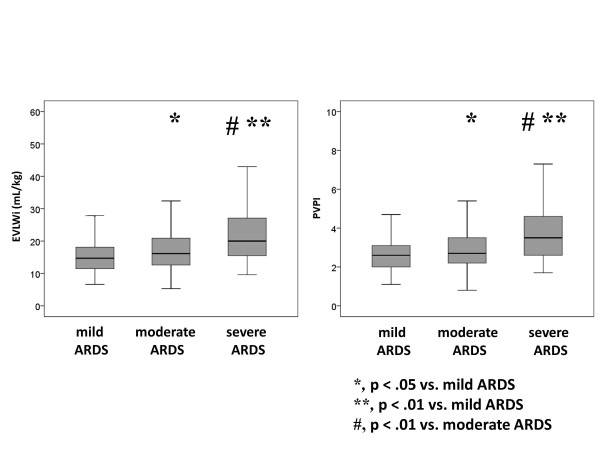
**Comparisons of the extravascular lung water index and pulmonary vascular permeability index measured using transpulmonary thermodilution among the three categories of acute respiratory distress syndrome**. Increasing ARDS severity was associated with higher EVLWi (mild, 14.7 (11.5 to 18.1); moderate, 16.2 (12.6 to 20.9); severe, 20.0 (15.5 to 27.3)) and PVPI values (mild, 2.6 (2.0 to 3.1); moderate, 2.7 (2.2 to 3.5); severe, 3.5 (2.6 to 4.7)). *P <0.05 vs. mild ARDS; **P <0.01 vs. mild ARDS; #*P *<0.01 vs. moderate ARDS. ARDS, acute respiratory distress syndrome; EVLWi, extravascular lung water index; PVPI, pulmonary vascular permeability index.

The results revealed negative and moderate correlations between EVLWi and the P/F ratio (r = -0.355, *P *<0.001) as well as between PVPI and the P/F ratio (r = -0.345, *P *<0.001) in all data sets (Figure 2). ARDS severity was associated with an increase in EVLWi (mild, 14.7 (11.5 to 18.1); moderate, 16.2 (12.6 to 20.9); severe, 20.0 (15.5 to 27.3)) and PVPI (mild, 2.6 (2.0 to 3.1); moderate, 2.7 (2.2 to 3.5); severe, 3.5 (2.6 to 4.7)) (Figure 3, Table 3).

**Table 3:** EVLWi and PVPI in patients with ARDS based on the Berlin definition during a three-day observation period.

	mild ARDS (*n *= 152)	moderate ARDS (*n *= 230)	severe ARDS (*n *= 113)
EVLWi, mL/kg	14.7 (11.5-18.1)	16.2 (12.6-20.9)**	20.0 (15.5-27.3)*#
PVPI	2.6 (2.0-3.1)	2.7 (2.2-3.5)**	3.5 (2.6-4.7)*#

*P <0.01 vs. mild ARDS; **P <0.05 vs. mild ARDS; #*P *<0.01 vs. moderate ARDS. ARDS, acute respiratory distress syndrome; EVLWi, extravascular lung water index; PVPI, pulmonary vascular permeability index.

## Discussion

In this study, we demonstrated that the Berlin definition of ARDS effectively predicts mortality, the severity of physiological dysfunction, and organ failure. When using the Berlin definition, ARDS severity is associated with an increase in EVLW and pulmonary vascular permeability as assessed using the transpulmonary single thermodilution method in patients who fulfilled the Berlin definition of ARDS with EVLWi ≥10 mL/kg.

The categories of mild, moderate, and severe ARDS as determined by the Berlin definition are associated with increased mortality and severity. The associated predictive validity for mortality is superior to that of the AECC definition. The relationship between ARDS stage and pulmonary microvascular permeability/EVLW content has not been evaluated, although increased EVLW content secondary to increased pulmonary microvascular permeability is widely considered to be a hallmark of ARDS [12].

The transpulmonary thermodilution single-indicator technique provides an estimation of both EVLW and PVPI [20]. This technique is as sensitive as the double-indicator technique and frequently used in clinical settings [17,18]. Previously, we validated the accuracy of EVLW measurements obtained using the single-indicator technique in postmortem lung samples and defined statistically normal EVLW values in a study of human cadavers [19]. The universal diagnostic criteria for ARDS cannot include the measurement of EVLW and pulmonary vascular permeability, because the procedures for measurement are invasive and not feasible for every institution. In this study, we evaluated the relationship among EVLWi, PVPI and ARDS stages in patients with an EVLWi of ≥10 mL/kg that was not fully explained by cardiac failure or fluid overload. Negative and moderate correlations were observed between the EVLWi and P/F ratio as well as between the PVPI and P/F ratio. Moreover, EVLWi and PVPI values increased in association with ARDS severity. These results suggest that the ARDS progression outlined by the Berlin definition is associated with increases in EVLW content and pulmonary microvascular permeability, which is the hallmark of lung pathophysiology. It has also been demonstrated that the Berlin definition distinguishes the severity categories of ARDS with good predictive validity for mortality, the severity of physiological derangements, and organ failure.

### Limitations

In this study, a subset of patients meeting the ARDS criteria with respiratory failure of more than five days has been excluded. However, the Berlin definition includes patients with respiratory failure within one week of new or worsening respiratory failure. Therefore, a subset of patients meeting the ARDS criteria as diagnosed by the Berlin definition with respiratory failure of more than five days were excluded from the study. The associated results may not fully reflect the characteristics and pulmonary pathophysiology of patients with ARDS as set out by the Berlin definition.

The mechanisms underlying respiratory insufficiency (for example, permeability, pulmonary edema, cardiogenic edema, and/or pleural effusion with atelectasis) were defined by expert consensus; hence, a subjectivity bias cannot be completely ruled out. Nonetheless, only patients who were considered eligible by all the experts were included in the final analysis.

Among 207 patients diagnosed with ARDS in a previous prospective observational study, 12 patients with PEEP <5 cmH_2_O but who did not require airway pressure release ventilation (APRV) were excluded. Patients managed using APRV with PEEP <5 cmH_2_O were included, because their airway pressure was kept higher during respiratory management. This means that some of the patients enrolled in this analysis did not completely fulfill the Berlin definition.

Pulmonary inflammation, that is, pneumonia, may affect the results obtained using the thermodilution technique. Inflamed cells and purulent matter, including microabscesses, might increase lung weight despite increased levels of EVLW. Further evaluation may be required to assess patients with ARDS secondary to direct injury caused by pneumonia.

An EVLWi of ≥10 mL/kg was used for defining pulmonary edema in this study, as reported previously. Although no definitive quantitative criteria for pulmonary edema were established, this value was selected on the basis of the value found in our recent human autopsy study and those used for defining pulmonary edema in previously reported studies. Lowering the EVLWi value for pulmonary edema could have led to the inclusion of patients with less severe pulmonary edema, which may have influenced the results.

In this study, each patient was managed with a PiCCO system for transpulmonary thermodilution monitoring of his circulatory/respiratory status. Therefore, patients with less severe ARDS may not have been selected for enrollment. Of 35 excluded patients, 14 were judged to have respiratory failure secondary to sepsis-induced increases in pulmonary vascular permeability but had to be excluded because they had previously presented EVLWi <10 mL/kg [21]. Nonetheless, there may exist a considerable number of patients meeting ARDS criteria with 'normal' EVLWi [38]. The inclusion and exclusion criteria used represent potential limitations of the study.

## Conclusions

The severity categories of ARDS laid out by the Berlin definition may be associated with increased EVLW and pulmonary microvascular permeability in patients who fulfilled the Berlin definition of ARDS with EVLWi ≥10 mL/kg, which is the hallmark of lung pathophysiology. Moreover, this staging has good predictive validity for mortality, the severity of physiological derangements, and organ failure. These relationships require confirmation through further clinical research and critical care practice.

## Key messages

- The categories of acute respiratory distress syndrome laid out by the Berlin definition may be associated with increased extravascular lung water and pulmonary microvascular permeability, which is the hallmark of lung pathophysiology.

- The categories of the Berlin definition of disease progression are also associated with the severity of physiological derangements and the incidence of organ failure.

## Abbreviations

AECC: American-European Consensus Conference; ALI: acute lung injury; APACHE: acute physiology and chronic health evaluation; APVR: airway pressure release ventilation; ARDS: acute respiratory distress syndrome; BNP: B-type natriuretic peptide; CT: computed tomography; EVLW: extravascular lung water; EVLWi: extravascular lung water index; GEDV: global end-diastolic volume; ITBV: intrathoracic blood volume; ITTV: intrathoracic thermal volume; LIS: lung injury scale; PEEP: positive end-expiratory pressure; P/F ratio: PaO_2_/FiO_2 _ratio; PTV: pulmonary thermal volume; PVPI: pulmonary vascular permeability index; SIRS: systemic inflammatory response syndrome; SOFA: sequential organ failure assessment.

## Competing interests

Dr. Taira is a member of the medical advisory board of Pulsion Medical Systems. The other authors declare no competing interests.

## Authors' contributions

All authors conceived and designed the study, wrote the study protocol, and acquired the clinical data. SK was responsible for the statistical analyses and the first draft of the manuscript. All authors amended and commented on the manuscript and approved the final version.
